# Economic and Environmental Assessment of Seed and Rhizome Propagated *Miscanthus* in the UK

**DOI:** 10.3389/fpls.2017.01058

**Published:** 2017-06-30

**Authors:** Astley Hastings, Michal Mos, Jalil A. Yesufu, Jon McCalmont, Kai Schwarz, Reza Shafei, Chris Ashman, Chris Nunn, Heinrich Schuele, Salvatore Cosentino, Giovanni Scalici, Danilo Scordia, Moritz Wagner, John Clifton-Brown

**Affiliations:** ^1^Institute of Biological and Environmental Sciences, University of AberdeenAberdeen, United Kingdom; ^2^Blankney Estates Ltd.Lincolnshire, United Kingdom; ^3^Bangor UniversityBangor, United Kingdom; ^4^Institute of Biological, Environmental and Rural Sciences, Aberystwyth UniversityAberystwyth, United Kingdom; ^5^Julius Kühn-Institut - Bundesforschungsinstitut für KulturpflanzenBraunschweig, Germany; ^6^Das Deutsche AgrarzentrumPotash, Ukraine; ^7^Dipartimento di Agricoltura, Alimentazione e Ambiente (Di3A), Università degli Studi di CataniaCatania, Italy; ^8^Institute of Crop Science, University of HohenheimStuttgart, Germany

**Keywords:** biomass, bioenergy, upscaling, GHG-cost, economic-costs, agronomy, seed-propagation, *Miscanthus*

## Abstract

Growth in planted areas of *Miscanthus* for biomass in Europe has stagnated since 2010 due to technical challenges, economic barriers and environmental concerns. These limitations need to be overcome before biomass production from *Miscanthus* can expand to several million hectares. In this paper, we consider the economic and environmental effects of introducing seed based hybrids as an alternative to clonal *M. x giganteus* (*Mxg*). The impact of seed based propagation and novel agronomy was compared with current *Mxg* cultivation and used in 10 commercially relevant, field scale experiments planted between 2012 and 2014 in the United Kingdom, Germany, and Ukraine. Economic and greenhouse gas (GHG) emissions costs were quantified for the following production chain: propagation, establishment, harvest, transportation, storage, and fuel preparation (excluding soil carbon changes). The production and utilization efficiency of seed and rhizome propagation were compared. Results show that new hybrid seed propagation significantly reduces establishment cost to below £900 ha^-1^. Calculated GHG emission costs for the seeds established via plugs, though relatively small, was higher than rhizomes because fossil fuels were assumed to heat glasshouses for raising seedling plugs (5.3 and 1.5 kg CO_2_ eq. C Mg [dry matter (DM)]^-1^), respectively. Plastic mulch film reduced establishment time, improving crop economics. The breakeven yield was calculated to be 6 Mg DM ha^-1^ y^-1^, which is about half average United Kingdom yield for *Mxg*; with newer seeded hybrids reaching 16 Mg DM ha^-1^ in second year United Kingdom trials. These combined improvements will significantly increase crop profitability. The trade-offs between costs of production for the preparation of different feedstock formats show that bales are the best option for direct firing with the lowest transport costs (£0.04 Mg^-1^ km^-1^) and easy on-farm storage. However, if pelleted fuel is required then chip harvesting is more economic. We show how current seed based propagation methods can increase the rate at which *Miscanthus* can be scaled up; ∼×100 those of current rhizome propagation. These rapid ramp rates for biomass production are required to deliver a scalable and economic *Miscanthus* biomass fuel whose GHG emissions are ∼1/20th those of natural gas per unit of heat.

## Introduction

Greenhouse gas (GHG) emissions to the atmosphere have to be curtailed so that global warming is limited to between 1.5 to 2°C as agreed in the 2015 Paris Agreement on climate change, signed by 197 countries and currently ratified by 142 countries including the largest GHG emitters, the United States and China. An important action to achieve this GHG emissions goal is to decarbonize the energy supply (Intergovernmental Panel on Climate Change [IPCC], 2014a,b). The key challenge is to achieve this equitably, without harming the economy or the environment. Nuclear and renewable energy systems powered by wind, solar, hydro, tidal, and biomass have low, but not zero GHG emissions per GJ of energy produced. They all require infrastructure to be built with energy intensive materials and have operating GHG costs, particularly where the production and distribution of energy vectors still rely on a fossil fuel based supply chains. There are also emissions associated with land use change, with indirect consequences that are difficult to quantify in many cases, although a methodology for annualized GHG emissions from carbon stock change due to land-use change has been developed by the Directive 2009/28/CE, and recently amended in the EU 2015/1513.

At present the economic cost per unit of renewable energy generated is more than that produced by systems powered by fossil fuels, though it should be noted that the lower price of fossil fuel is largely determined by huge, and largely hidden, national and global subsidies for production and use, totalling between 0.5 to 2 trillion US dollars per year (World Bank, 2015). Renewable energy systems also currently rely on subsidies in various forms to support their production costs. Historically in the United Kingdom, these take the form of the United Kingdom’s Renewable Obligation Certificates (ROCs), Feed in Tariffs (FIT), and Renewable Heat Incentives (RHI) (Department of Energy and Climate Change [DECC], 2013). The most recent for electricity generation is the Contracts for Difference (CfD) whereby the generator is guaranteed a wholesale price per MWh of electricity generated, called a Strike Price (Ofgem, 2016). The current strike price levels vary with technology and are significantly above the current range of wholesale prices per MWh for fossil and old nuclear electricity (Ofgem, 2016), which averages around £40 MWh. As examples, strike prices for the period 2016/17 are: anaerobic digestion – £150, dedicated biomass with combined heat and power (CHP) – £125, hydro – £100, onshore wind – £95, offshore wind – £155, large scale solar – £115 and tidal stream – £305. For comparison new nuclear has a strike price of £93 MWh for the first facility.

The current wholesale price for electricity includes carbon tax and the cost of fuel and so subtracting these the generators get around £20 MWh to cover operating costs (OPEX) and the amortization of capital expenditure (CAPEX). It is therefore clear that without taxes on carbon and generation subsidies, renewables are not currently economically competitive. In order for these renewable energy systems to be used to reduce GHG emissions in an economically viable way, it is important to quantify the economic and environmental costs of energy production for a range of technological options.

Bioenergy systems are one form of renewable energy. After centuries of burning wood for energy or processing forage into horse power, the first generation of bioenergy feedstocks were food crops, such as maize, oil seed rape, sugar cane, and oil palm, used to produce bioethanol and biodiesel. These required a high input in terms of fertilizer and energy, which increased their carbon footprint (St. Clair et al., 2008). In addition, the carbon cost of converting the food crop feedstock to bioethanol or biodiesel was significant with a low ratio of energy produced to energy input, high GHG cost and a low productivity in terms of GJ of energy per hectare of land (Hastings et al., 2012). Another drawback of using food crops for energy production is the pressure put on the balance of supply and demand for these feedstocks which can impact the cost of food (Valentine et al., 2011) and the increase of indirect land use change (ILUC) to increase the arable cropped area (Searchinger et al., 2008) which consequentially increases their environmental footprint.

The second generation bioenergy crop *Miscanthus* almost always has a smaller environmental footprint than first generation annual bioenergy ones (Heaton et al., 2004, 2008; Clifton-Brown et al., 2008; Gelfand et al., 2013; McCalmont et al., 2015a; Milner et al., 2015). This is due to its perennial nature, nutrient recycling efficiency and need for less chemical input and soil tillage over its 20-year life-cycle than annual crops (St. Clair et al., 2008; Hastings et al., 2012). *Miscanthus* can be grown on agricultural land that is economically marginal for food crop production (Clifton-Brown et al., 2015). However, the planted area of *Miscanthus* for biomass in Europe has stagnated since 2010 for a range of reasons including technical challenges, economic barriers, and environmental concerns. *Miscanthus* is in the early stages of domestication (Clifton-Brown et al., 2015) and poor agronomy of many of the first crops planted by rhizome propagation resulted in patchy establishment and consequent yield losses (Zimmerman et al., 2013). This was further compounded by the ending of incentive schemes such as the United Kingdom Energy Crops Scheme (which closed in 2013 for new applications) and uncertain markets (e.g., Drax power station withdrew from burning *Miscanthus* in 2016 on the announcement of significant reductions in government support (Farmers weekly, 2016)). There are some signs of recovery in the United Kingdom biomass market with new dedicated straw burning power stations, such as Brigg in Lincolnshire (taking some 25,000 tons of *Miscanthus* per annum), coming online and providing some market pull. However, significant uncertainty remains in the market place making the decision by farmers and land owners to grow *Miscanthus* difficult, as the land must be committed to the perennial crop for its 10- to 20-year economic lifetime. Unlike annual arable crops, farmers cannot maximize farm profitability by changing crop species each year to follow market prices; highly front loaded perennial crop establishment costs require a long-term market to return the investment.

Certainty in crop establishment is important to avoid unwanted planting gaps, patchiness and the resultant yield losses which persist for the lifetime of the crop. There is a need to accelerate stand establishment in cool temperate climates to minimize the time to achieve maximum economic harvest, which in the historical plantings was about 3–5 years (Lesur et al., 2013; Clifton-Brown et al., 2015) depending on the local environmental conditions. As the crop is established once in its lifetime of up to 20 years, the cost of establishment has to be amortized over its entire lifetime. This means that the actual cost of production per ton, in terms of its GHG, energy-use or monetary cost, is increased if the crop fails or is slow to reach full productive potential due to poor establishment.

Current development of techniques to introduce new and seed propagated hybrids of *Miscanthus* with the associated novel agronomies and developments in harvesting (Clifton-Brown et al., 2016) have been projected to make significant reductions in the cost of producing and processing the biomass for end uses. Here, we make an experimental and modeling assessment to quantify how technical developments impact the economic and environmental performance of the crop using several trials, including those from the OPTIMISC (Lewandowski et al., 2016) and GIANT-LINK projects (Clifton-Brown et al., 2016), of which the ‘Blankney large scale seed trial’ (5 ha, planted in 2012) was a part and is a back bone of this study. This was the first trial of its kind, creating essential knowledge critical to move *Miscanthus* from a clone based crop to one based on seed. Other field scale experiments, such as the 6 ha trial planted in Penglais (Aberystwyth) in 2012 for the Carbo-biocrop/ELUM projects^1^^,^^2^ were also used (McCalmont et al., 2015b; Dondini et al., 2016). Subsequently, field trials, sized to be commercially relevant, were planted with improved hybrids and agronomies in 2013 near Stuttgart (Germany), Potash (Ukraine) and the United Kingdom (three sites) (unpublished data). Data from a further four sites planted in 2014 in the United Kingdom with newer hybrids produced during GIANT-LINK show that seeded genotypes are matching yields of *Mxg* (unpublished data). The trials used in this study and their relevant details are shown in **Table 1**. The setups for these trials are described in Clifton-Brown et al. (2016), Lewandowski et al. (2016), and Kalinina et al. (2017).

**Table 1:** Field trials used in this study with details of the propagation and harvesting methods used.

		Propagation methods				Harvesting methods						
Name	Planting year	Rhizome	*In vitro* plugs	Seed plugs	Film	Hand	Direct chip	Mow	Swath drying	Bale	Pellet	Reference
Blankney large scale (5 ha), United Kingdom	2012	√		√			√	√	√		√	Lewandowski et al., 2016
Penglais (6 ha), United Kingdom	2012	√					√					McCalmont et al., 2015b
Multi-location GXE trial OPTIMISC	2012		√	√		√						Kalinina et al., 2017
Lincoln commercial planting, United Kingdom	2012–present	√						√	√	√	√	Unpublished M. Mos
Ihinger Hof large scale (0.6 ha), DE	2013			√			√					Unpublished T. Truckses
DAZ large scale (2 ha), UA	2013			√			√					Unpublished H. Schuele
Multi-location GIANT LINK hybrid trial, United Kingdom	2014	√		√	√	√						Unpublished J. Clifton-Brown
Film effects plot trial, United Kingdom	2014	√		√	√	√						Unpublished C. Ashman
GIANT Elite seed trial, United Kingdom	2015	√		√	√	√						Unpublished R. Shafiei
Unterer Lindenhof large scale GNT	2015	√		√	√			√				Unpublished A. Kiesel

We report on measurements of the energy, carbon intensity and economic cost of each phase of *Miscanthus* production, from propagation to final biomass fuel preparation, made on field scale experiments in the United Kingdom and Europe. We have used a sensitivity analysis to identify critical foci for further research efforts to make biomass systems an economic and environmental alternative to fossil fuel energy systems.

## Materials and Methods

This study used data from the *Miscanthus* crop trials detailed in **Table 1** as well as measurements made on commercial *Mxg* plots in the United Kingdom to examine the economic cost and GHG emissions of each component of the processes used to produce *Miscanthus* biomass fuel. The use of many experiments was necessary as individual agronomy tests require up to 4 years to produce results and many of the experiments were conducted in parallel, in multiple locations. For each production process the trial used is identified and the evaluation methodology described. Additional information from the literature was used for aspects of production not tested.

### Units, Parameters, and Criteria Used

The actual costs in pounds sterling (GBP) at the time of the experiment in 2015 is used, references to cost in other studies are referred to in their quoted currency and converted to pounds sterling at an exchange rate of £1 = 1.2 and £1 = $1.26. Economic costs involving the use of machinery are based on 2015 market equipment rental rates in the United Kingdom and 2015 fuel prices, based on $50 per barrel of oil with United Kingdom retail prices of £0.80 l^-1^ for diesel and £0.13 kWh for electricity. Transport distances are in United Kingdom miles (eq. to 1.61 km). Harvests are reported in Mg (metric ton) and commercial harvest yields are derived from counting bales and multiplying by the average bale weight for the genotype and correcting for moisture content using measurements from on-farm moisture gauges used for straw and grain. Only the operational costs of producing *Miscanthus* were quantified in this study as these can be related to the operational costs for producing other crops, this means the overhead cost of land and buildings, which vary by county, were not considered.

The higher heating value of *Miscanthus* was taken as 18 GJ Mg^-1^ harvested dry matter (DM; Sims et al., 2006). GHG emissions are expressed as the amount of CO_2_, N_2_O, and CH_4_ converted to their global warming potential (GWP) over 100 years as “equivalent CO_2_,” using CH_4_ = 34 CO_2_ and N_2_O = 298 CO_2_ (Intergovernmental Panel on Climate Change [IPCC], 2014a). GHG quantity is expressed as kg of C in the equivalent CO_2_ (CO_2_ eq. C). This is used to define the GHG emitted per MJ of energy (g CO_2_ eq. C MJ^-1^) in the crops energy or mass of crop (g CO_2_ eq. C Mg^-1^). A similar unit is used to define the embedded GHG’s in machinery and fuel used per Mg of biomass harvested or ha of land worked upon. Machinery GHG cost is calculated from the United Kingdom average of 55 kg of machinery ha^-1^ and based upon a 10 year service life, this is 13 kg CO_2_ eq. C ha^-1^ y^-1^. Electricity emissions are United Kingdom national grid average 31.9 g CO_2_ eq. C MJ^-1^ based on the 2015 generation mix (Digest of UK Energy Statistics, and Electricity Statistics [DUKES], 2016). GHG emissions from diesel fuel is 0.86 kg C kg^-1^ diesel.

Soil organic carbon changes (SOC) were not measured or considered in this study as the time period to observe change (1–3 years) was too short in the trials used. Although SOC changes under *Miscanthus* have been measured for rhizome propagation (Dondini et al., 2009; McCalmont et al., 2015a,b; Dondini et al., 2016) and the impact modeled spatially (Milner et al., 2015; Pogson et al., 2016; Richards et al., 2016), the impact of seed propagation on SOC has not yet been evaluated.

The use of fertilizers is not considered in this study as they have not been used on the commercial plots in the trial, nor currently in commercial plantings in the United Kingdom which rely on the initial nitrogen, phosphorous, and potassium (NPK) load of arable or rotational pasture field, which is normally sufficient for successful *Miscanthus* crop establishment (Michal Mos, private communication). In addition previous experiments indicate that for the management considered in this study, spring harvests after plant senescence, N fertilization has little impact on yield due to the low take off at harvest (Clifton-Brown et al., 2007; Davis et al., 2010).

The yields used in the calculation of GHG emissions and crop economics this study used mean yields of 12–14 Mg ha^-1^ y^-1^ that have been observed from *Mxg* from current commercial plantings observed in the United Kingdom (private communication, M. Mos). We have assumed a logistic yield increase for establishment year yields and a linear decline in yield after 15 years Lesur et al. (2013). Inter-annual yield variation, due to weather conditions, as observed in long term trials (Clifton-Brown et al., 2007) and modeled *Miscanthus* yields for the United Kingdom, using weather data from 2000 to 2009 (Harris et al., 2014) using the MiscanFor model (Hastings et al., 2009, 2013) indicates that the weather related standard deviation of inter-annual yield variation in the United Kingdom is of the order 2.1 Mg ha^-1^ y^-1^ for a mean yield of 10.5 Mg ha^-1^ y^-1^ for the whole of the United Kingdom. The modeled yields are generally pessimistic as they calculate rain-fed yields and do not account for ground water support that is available in many United Kingdom arable farms.

### Statistical Tests

Minitab 17 software (Minitab, Inc., State College, PA, United States) was used to conduct the data exploration, data conditioning and analyses. Descriptive statistics were used to calculate means and standard deviations of the tests and comparisons between treatments were made by one way ANOVA using the Tukey HSD test (*P* < 0.05).

### Field Trials Used

The Blankney Trial was the first large commercially relevant scale *Miscanthus* trial in the United Kingdom using seeds. It was part of the proposal of both the United Kingdom and EU funded projects, GIANT-LINK^3^ and OPTIMISC^4^, respectively. The trial was located at Blankney, Lincolnshire. The objective was to raise sufficient seed to plant 4 hectares (at 20,000 plants ha^-1^) to compare with *Mxg* planted from rhizomes (at 16,000 plants ha^-1^). The trial is described as WP 5 in Lewandowski et al. (2016). This trial was used for the plug production, weed control and harvesting experiments and provided material for the pelleting trials. It should be noted that the seed propagated clones were not chosen for their high yield but the ability of the parents to produce a sufficient quantity of seed to make the required agronomy tests.

The Penglais commercial-scale trial of *Mxg*, described by McCalmont et al. (2015b), was used to test yields in the Atlantic seaboard maritime climate, direct chipping and provided material for animal bedding trials.

The OPTIMISC multi-location, multi-hybrid trials described in Lewandowski et al. (2016), Kalinina et al. (2017), and Nunn et al. (in press) were used to determine yields of 15 germplasm types (of which 11 were clonal genotypes, and 4 from seed) in six contrasting soil and climatic conditions distributed in western Eurasia. The propagation of the clonal types was used to estimate the cost of *in vitro* plug production.

The commercial plantings managed by Terravesta Ltd. in Lincoln, United Kingdom were used to estimate the cost of preparing the soil for planting, production, storage, and planting of rhizomes and cutting and baling techniques to optimize *Miscanthus* fuel quality (M. Mos private communication).

Two large scale trials set up in OPTIMISC program in Stuttgart and Ukraine were used to trial establishment of seeded hybrids, weed control and commercial yields (Lewandowski et al., 2016) (private communication A. Kiesel).

The GIANT-LINK program multi-genotype replicated trials were used to test the yield of many novel seed based and clonal hybrids in contrasting locations (United Kingdom, Poland, Ukraine, and Germany), using *Mxg* as a comparison. These identified several new hybrids that had yields greater than or equal to *Mxg*. In addition different agronomies for direct seeding, plug planting and the use of mulch film were trialed and the processing of seed tested (private communication, J. Clifton-Brown).

The film effects trials in Aberystwyth and Hackthorn (United Kingdom) were used to measure the effect on crop establishment for direct seed, seed and plug and rhizome propagated plants. The trials were replicated with each of the different propagated methods being trialed with and without mulch film. The yields were measured for 2 years, here the comparison of *Mxg* and seed plug establishment is reported (paper in preparation, C. Ashman and D. Awty-Carroll).

The GIANT Elite seed trial, was similar to the GIANT-LINK and OPTIMISC multi-hybrid trial but tested new hybrid material that was bred during the GIANT-LINK project. Its primary purpose was to test yield performance and establishment rate in two contrasting locations. Twelve inter-species hybrids (designated GNTxx) were seed plug propagated and planted in triple replicated trials under mulch film in Oxford, United Kingdom and the Julius-Kuehn Institute (JKI) in Braunschweig in Germany. The plot sizes were 50 plants at a commercial size spacing. The trial is ongoing and the results presented here relate to the second year harvest (Private communication GIANT-LINK team).

### Crop Establishment: Soil Preparation, Weed Control, and Mulch Film

Crop establishment has four cost components: creation of the plant material, soil preparation, planting, and weed control. The economic and GHG costs of each component were determined using measurements from the Blankney trials and commercial planting experience by Terravesta Ltd. The machinery used, the time and fuel consumption for each operation per hectare were measured or estimated. From this information the GHG emissions were calculated.

Soil preparation and weed control are site specific and depend on the initial land use and vegetation, ecology, soil texture and drainage and climatic conditions, which will govern the machinery, fuel and products used. It is important to reduce C_3_ weeds as these emerge early and can out compete young *Miscanthus* plants. To date, it is normal to use Glyphosate [*N*-(phosphonomethyl)glycine] several weeks before plowing to remove the previous crop and or weeds. The soil is normally inversion plowed, though low-till methods are also possible. Just before planting, the soil is worked to a fine tilth with a tine or power harrow. For heavier and or marginal soils, two or more passes of a power harrow may be needed to prepare a fine tilth. The soil preparation input requirements depend on site/soil conditions and are the same whichever method of planting is used (rhizomes or plugs). During the first growing season, weeds were controlled to ensure minimal competition using a Jubilee (200 g/kg metsulfuron-methyl) + Starane (100 g/l fluroxypyr + 2.5 g/l florasulam) mix. Only one application is usually required with a GHG cost of 7 kg CO_2_ eq. C ha^-1^. This would be multiplied by the number of applications in the case of severe weed infestation. The base case ground preparation considered here is moldboard plowing and disk and tine harrowing with GHG costs of 165 kg CO_2_ eq. C ha^-1^, in heavier soils that require sub-soiling and more passes of the power harrow, this value would double.

The trial of the use of mulch film in Aberystwyth was used to test different crop establishment agronomy when combined with under-film weed control on seed, plug and rhizome plantings. The yield was measured over 2 years and compared to a control of no film for all treatments. *Mxg* was used as a comparison. The difference in yields between treated and control plots of *Mxg* were tested by ANOVA. Film costs £100 per ha and GHG emissions from its manufacture and application are 220 kg CO_2_ eq. C ha^-1^.

### Rhizome Propagation

The *Mxg* rhizomes used in this experiment were produced in the United Kingdom. Producing rhizomes for propagation in the United Kingdom climate takes at least two growing season, this entails clearing the production ground of weeds, plowing in spring and tilling the ground to a fine seed bed like tilth before planting the rhizomes with a potato type planter. During the growing season weeds were controlled to ensure minimal competition using Jubilee + Starane mix. In the spring following the second growth year, the rhizomes are harvested using a modified potato harvester, hand or semi-automatically sorted and cut into viable pieces, 20–40 g. Harvested rhizomes are moved the same day to cold storage (2–5°C) before being transported to the crop planting site just before use to ensure the highest possible rhizome viability to maximize the establishment rates. This is typically 80–90% with fresh rhizomes planted within 2–3 days of harvesting or those kept in cold storage for longer periods (Terravesta, personal communication). One ha of rhizomes produces enough material to plant 10–30 ha of crop with the same modified potato type planter. Lower quality rhizomes, tested by sprouting tests, would require 80–90 g rhizomes (private communication, M. Mos). The above ground biomass of the first growing season is mulched in the following spring and left as a soil amendment and for the second growing season is either harvested or mulched prior to rhizome harvesting. The economic and GHG cost of each component of production were calculated using the methods in “Crop Establishment: Soil Preparation, Weed Control, and Mulch Film” Section. If the rhizomes are produced in an environment where plant establishment is faster such as Poland, then the rhizomes can be harvested after the first growing season reducing the cost and improving the propagation rate. Here, the costs of United Kingdom rhizome production are considered.

### *In Vitro* Micro Propagation

*In vitro* propagation is a skilled and labor intensive activity where clone growth is achieved by *in vitro* tillering on a suitable sterile medium (Lewandowski and Kahnt, 1993). *In vitro* tillers are split approximately monthly by hand under sterile conditions (private communication, K.-U. Schwarz). When the required numbers of clones are reached, the tillers are transferred out of sterile conditions into peat soil and grown in a glasshouse for 8 weeks until viable rooted plantlet plugs are achieved. This method can also be used to produce parent plants for seed production. The costs considered include the laboratory manipulation space, equipment and human resources and the greenhouse space and heating. The plugs are planted either by hand or using a standard Checchi and Magli Trium plug planting machine.

### Seed Production, Direct Seeding, and Seed-Plug Propagation

The GIANT-LINK project funded by UK’s DEFRA and BBSRC (2011–2016) in collaboration with CERES Inc. has successfully bred scalable seed propagated interspecies hybrids since 2013. Agronomic trials have shown, while successful establishment by direct sowing is possible, current methods waste seed and are often unreliable. To reduce the risks, the strategic emphasis has been on planting seeded hybrids via plug plants into the field (Clifton-Brown et al., 2016).

Seed production has to be conducted in climatic environments where the parental lines flower, cross pollinate and produce seed each year. Seed used in the Blankney large scale trial planted in 2012 was produced through open pollination of selected breeder’s lines flowering in Braunschweig (Germany). Seeds from controlled field pollination from specifically planted ‘crossing blocks’ in Texas, (United States) and Sicily (Italy) were produced from 2013 onward. Seed production requires intensive management. The pollen and seed parents are cloned from ‘mother plants’ either by splitting rhizomes or *in vitro* tillering. The parental cloning rate depends largely on the parental species in question. In dry periods irrigation management is the key to successful seed set. Seed from ripe panicles in autumn is threshed and cold stored. Seed germination rates vary due to many factors including ripeness at harvest and dormancy (Awty-Carroll, 2017).

Our work is showing, depending on the hybrid type, one ha of seed production can produce enough seed for ∼1000–2000 ha of planting, depending on parental combinations, two orders of magnitude greater than rhizome propagation. The economic and GHG costs for all of the operations required for seed production added together are high due to the labor intensity of the agronomy. However, the cost of production is divided by the number of ha to be planted.

Trials with direct drilled *Miscanthus* seed trials are ongoing in the United Kingdom with an adapted Agricola Italiana precision pneumatic seed drill [35010 Massanzago, (PD), Italy] and have been shown to be a viable option of propagation. The longer term objective is to make *Miscanthus* seed drilling routine, though many barriers still exist (Ashman and Awty-Carroll, personal communication). Here, the GHG and economic costs of direct seeding *Miscanthus* have been estimated using current protocols for farm operation with direct seeding based upon a seed drill being pulled by a tractor with a driver and one other operator.

**FIGURE 1:**
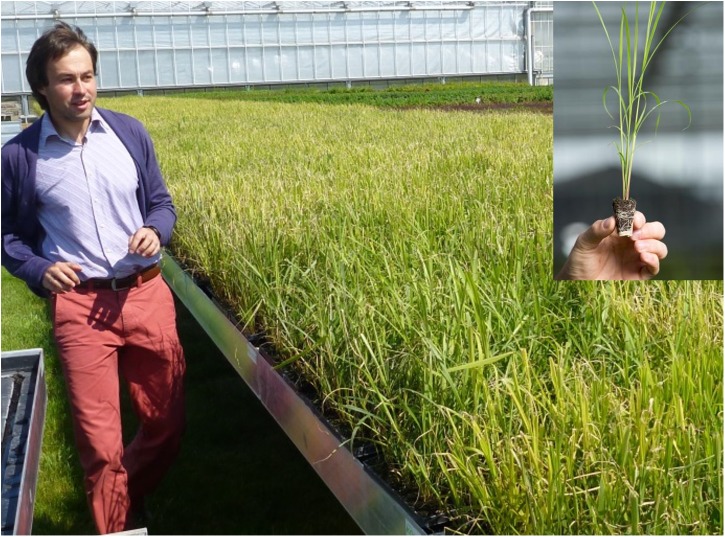
Eight-week-old seed established plug plants produced in peat soil in multi-trays in the glasshouse, hardening pre-planting. Inset shows a single plug ready for planting. Main picture of the multi-trays was provided by Dr. Michal Mos who features in the picture and has consented to his picture being published, inset picture of the plug was provided by John Clifton-Brown.

Technology for the plug production from seeds has been developed by Bell Brothers Nurseries Ltd. (United Kingdom), employing techniques used in the horticulture of vegetables and in field establishment agronomy using plug planters and film developed by IBERS/Terravesta Ltd. (United Kingdom) so that an 85–95% establishment rate is achieved. The seeds are planted in modules in a glasshouse around 8 weeks before field planting. Timing of the planting date affects the energy used in the greenhouse. Earlier sowings in January require more glasshouse heating than later sowings in early March (**Figure 1**). The cold hardened plugs are planted into a fine tilth to ensure good plug to soil hydraulic contact by a standard Checchi and Magli Trium plug planting machine. Economic and GHG costs were calculated using time and space estimates from the nurseries and the costs of standard farm machinery. This requires a tractor pulled planter with one operator per two rows planted. Currently, as is common practice in the horticultural industry, following personnel (one per four rows) heel in any missed plants.

### Harvesting Tests

In the spring following the third growing season after planting, direct harvesting with a forage harvester was compared with the indirect harvest method using a mower and baler at the Blankney site. Biomass from both methods was used to make 6 mm pellets (Farm Feed System, United Kingdom). Direct chipping was also used at the Penglais site. The choice between chipping and baling will depend on the end use required, the storage available and the transportation distance to the end user. Both methods were evaluated at the Lincolnshire (United Kingdom) site to determine and compare the timing of each operation, the fuel consumption and the cost.

Direct chipping was evaluated on the 2015 harvest of three replicate plots of an open pollinated *M. sinensis* hybrid (OPM12) at Blankney in Lincolnshire on a scale which is representative of commercial fields. Three plots were mown with a forage harvester fitted with a 7.5 m wide cutter and chipped into a following trailer. The plots were 54 m long and mown in 7.5 m passes. The time to cut and chip each of several passes for each replicate was recorded as well as the fuel consumption and the biomass harvested. Machinery used was a Claas Jaguar 859 with a 7.5 m Claas Orbis header, a Claas Arion 650 tractor (184 hp) with a Baily silage trailer, this was operated by two staff. For commercial operations on large fields at least two tractors with silage trailers operated by an additional person would be required for continuous operation. The fuel consumption, timing and cost of each operation were recorded. From this the GHG cost was estimated.

Cutting to swath and baling was evaluated on the 2015 harvest of three replicates plots of three open pollinated breeder’s lines (OPM52, OPM53, and OPM54) and *Mxg* (OPM9) at Blankney in Lincolnshire. These hybrids differ in leaf share and stem diameter, with the classic antagonistic relationship between stem counts per plant and height (Kalinina et al., 2017). The plots were cut to a swath using a 4.5 m mower using three 54 m runs. The time to cut a swath for each of several passes for each replicate was recorded as well as the fuel consumption and the biomass harvested and the results aggregated to hybrid means. The machinery used for mowing to a swath was a Claas Jaguar 859 with a 4.5 m Claas RU450 header (Claas, Harsewinkel, Ostwestfalen-Lippe, Germany); as the crop was sufficiently dry this was immediately followed by a Fendt 720 tractor (184 hp) with a Massey Ferguson MF2290 120 x 120 x 240 Hesston baler and chased by a JCB Loadall 531-70 telehandler (J C Bamford, Rocester, Staffordshire, ST14 5JP) and a Claas Arion 650 tractor with a Baily flat-bed trailer to transport to the store. This was operated by four staff. For continuous operation on a large field at least two tractors with flat-bed trailers operated by an additional person are required. If turning of the swath on the field is required to dry the crop it can achieved at the rate of 2 ha h^-1^ with a 150 horse power tractor and a hay turner. The crop is normally dried in the swath until the moisture is below 14%. The fuel consumption, timing and cost of each operation were recorded. From this the GHG cost was estimated.

### Pelleting

Pelleting *Miscanthus* biomass involves taking the feedstock, either from bales or chips and chipping it to <100 mm and then grinding it to <5 mm before pelletization. In this experiment, a Timberwolf chipper (TWSX200DHB, Stowmarket, United Kingdom), knife mill (SM 2000, Retsch, Haan, Germany) and MiniPress Pellet Mill (Farm Feed System, Cinderford, United Kingdom) were used to compare the ‘pellet-ability’ of a variety of *Miscanthus* genotypes and to estimate the energy required and conditions required to achieve useable pellet fuel.

The hybrids chosen for this pelletization experiment covered the range of plant morphologies observed in the *Miscanthus* hybrids. The energy to mill and pelletize was recorded and the GHG cost estimated from the energy consumption (United Kingdom grid electricity at 31.9 g CO_2_ eq. C MJ^-1^). It is important to note that as cost and energy-use is scaled with the size of the pellet mill, this experiment can only be used to compare the differences between hybrids. The economic costs and energy-use of pelleting commercial *Mxg* was obtained from commercial pelleting mills of differing sizes [e.g., La Meccanica CLM200, 15 kW, Italy, Pellet Mill in Condex Ltd. (Lancaster, United Kingdom)].

### Transport Costs

Using standard United Kingdom costs of truck transportation and normalized fuel consumption for the types of vehicle used (Department for Transport, 2014), the GHG and economic costs of transporting *Miscanthus* chips, bales and pellets was calculated. This was used along with the costs of chipping, baling and pelletizing to build a model to estimate the optimum distance between field, pellet mill and end user for each feedstock to minimize cost and GHG emissions. It was also used to optimize the trade-offs of transport costs with feedstock type for pelleting.

### Farm Profitability

A farm economic model was constructed in Microsoft Excel to estimate the relative profitability of *Miscanthus* crops for different yields and establishment rates grown with various crop establishment, management, and harvest methodologies to estimate their impact on the return on investment to the farm.

The analysis is based on a 150 ha farm with 10% *Miscanthus*. The model assumes that *Miscanthus* reaches peak yield after *x* years (*x* variable in the model) and is productive for up to 20 years. The establishment rates and harvest yields were taken from the OPTIMISC multi-hybrid trials (Kalinina et al., 2017; Nunn et al., in press), where peak-yield took between 1 to 4 years, depending on soil and climatic conditions. The costs of establishment, crop management, and harvesting are from the experiments reported here. Yield evolution assumes that peak production continues until year 15 after which it declines by 5% per year until year 20 (Lesur et al., 2013; Arundale et al., 2014a).

The model uses an amortization period of 20 years to coincide with the economic life cycle of *Miscanthus* and discount rate of 6% (variable in the model) is assumed for the comparisons. A *Miscanthus* feedstock farm gate selling price of £88 Mg^-1^ DM was used to calculate crop income, which was based on the current United Kingdom price for bales with 15% moisture of £75 Mg^-1^. All these values can be varied in the model to test sensitivities and to compare to other economic scenarios, however, the values used in our example reflect United Kingdom economic conditions and crop management in 2016. Land and buildings value was not considered in this study as it is site specific.

Scenarios tested were a comparison of rhizome and seed establishment and harvesting using either chipping or baling in Aberystwyth (United Kingdom), Potash (Ukraine), and Stuttgart (Germany). For each we calculated the gross margin and cumulative gross margin or net present value (NPV). Cumulative gross margins at zero indicates the break-even year on the graphs. Results for these scenarios are tabulated (**Table 9**).

## Results

### Crop Establishment Costs

**FIGURE 2:**
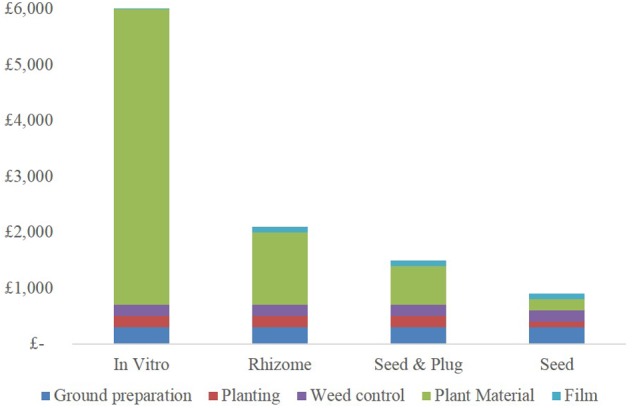
Estimated components of economic cost of establishing one ha of *Miscanthus* using *in vitro*, rhizome, plugs from seed and direct sown seed propagation. Components include ground preparation, planting, weed control, planting material, mulch film. Here only a single pass of the power harrow is considered to produce a fine soil tilth (*in vitro* projects the cloning of 1 hectare’s worth of material from a single clone which is being used as a parent for seed production).

Of the four components of crop establishment mentioned earlier, soil preparation and weed control are site specific, determining the types of machinery, fuel and herbicides used. **Figure 2** shows the base case of light soil with moderate weed control. The ground preparation, weed control and use of film is the same for each propagation method. The main establishment cost variable is the type of material planted as shown in **Figure 2**. *In vitro* is the most expensive followed by rhizome, seed and plug and the lowest is direct seed drilling. The specific cost of rhizome and plug planting are similar as they are relatively labor intensive whereas seed drilling, is predicted to halve the cost. The overall cost of plug propagation is 2/3 that of rhizome due mainly due to the higher multiplication factor of ∼2,000 to 1 compared to rhizome of 10–30 to 1. Direct seed drilling halves the cost of *Miscanthus* establishment (compared to rhizomes) to below £900 as well an increasing the ability to ramp up planted acreage. Even greater ramp ups can also be achieved by seed-plug propagation because less seed is wasted.

Greenhouse gas cost is broken down into the economic categories plus machinery manufacture as shown in **Table 2**. Weed control assumes only one application in the first year at 7 kg CO_2_ eq. C ha^-1^, and the base case of moldboard plow and disk and tine harrowing and costs 165 kg CO_2_ eq. C ha^-1^. Planting material has the largest variation. *In vitro* costs are much higher due to the intensive use of controlled environments in the early stages of cloning. Rhizome costs are a little higher than seed but seed-plugs are 4× that of rhizomes due to the use of greenhouse space for their production.

**Table 2:** Establishment greenhouse gas costs per hectare, to be amortized over total *Miscanthus* yield over the crop lifetime yield.

	GHG cost kg CO_2_ eq. C ha^-1^			
	*In vitro^∗^*	Rhizome	Seed to plug	Seed to soil
Plant material	18,905	49	1,264	0.1
Ground preparation	165	165	165	165
Machinery manufacture	13	13	13	13
Planting	233	265	233	233^∗∗^
Weed control	7	7	7	7
Total	19,323	499	1,682	418

*^∗^Refers to providing 1 ha of plug plantlets from one plant*.

*^∗∗^Assumes use of the same horsepower tractor as the plug planter*.

### Mulch Film Trial

The mulch film trial in Aberystwyth showed a significant (*P* < 0.05) difference between establishment rates for varying plant densities with the cumulative first 2-year mean yield almost doubling under film as shown in **Table 3**. Using film adds £100 per ha and 220 kg CO_2_ eq. C ha^-1^, to the cost of establishment. The effect of this increase is to reduce the establishment period of the crop by 1-year in Aberystwyth environmental conditions, similar reduction in establishment times were observed at the other trial sites and also in Ireland (O’Loughlin et al., 2017).

**Table 3:** Two-year dry matter (DM) yield comparison of *Mxg* with/without film in Aberystwyth film effect plot trial.

Treatment	Replicates	2-year DM yield (Mg ha^-1^)		Tukey group
		mean	SD	
Film	9	7.31	2.36	a
No film	9	3.96	1.13	b

### Harvesting by Chipping

Direct chipping results are tabulated in **Table 4**. The fuel consumption was 26 l ha^-1^ and with an average yield of 5.7 Mg DM ha^-1^, the diesel consumption was 4.56 l Mg^-1^. The rate of harvesting with this technique averaged 4.1 ha h^-1^ or 28 Mg h^-1^. In this experiment, the moisture content was 15% and the density of the chipped material was estimated to be 80–100 kg m^-3^. As the yield of this genotype was low and chipping rate is a function of crop throughput, the cost was calculated on a per Mg basis. The cost of this operation for large fields using two tractors and silage trailers is £28 Mg^-1^. The GHG emissions including fuel use and carbon (C) embedded in the machinery (which is 7.27 kg CO_2_ eq. C Mg^-1^). This estimate is for a yield of 5.7 Mg (DM) ha^-1^ which will change if the chopper has to work harder with thicker stems or a heavier (taller) crop.

**Table 4:** Harvesting *Miscanthus* (hybrid OPM-12) using direct chipping.

Number of 54 m × 7.5 m cutting ‘runs’	Average time taken per ‘run’ (s)	Standard deviation time taken per ‘run’ (s)	Sum area of runs = total sampled area (ha)	Diesel used for sampled area (l)	Mass from the total sampled area (Mg)	Harvest speed (ha h^-1^)	Harvest speed @15% moisture (Mg h^-1^)
12	35.4	2.7	0.49	12.6	3.3	4.1	28.0

### Harvesting by Cutting to Swath and Baling

The time to cut to a swath for each of several passes for each replicate was recorded as well as the fuel consumption and the biomass harvested. The mean results for each hybrid are tabulated in **Table 5**. The time to cut each swath run varied by 37% with the yield and hybrid (stem thickness), but at the slowest rate, a harvest rate of 23.3 Mg DM h^-1^ could be achieved on a thick stemmed hybrid like *Mxg*. The fuel consumption was estimated to be 10 l ha^-1^ with the 4.5 m cutter. The yield varied between 6.3 and 12.8 Mg DM ha^-1^, so the diesel consumption varied between 1.59 and 0.79 l Mg^-1^. The rate of cutting with this technique averaged 2 ha h^-1^ or 12.1 to 23.3 Mg h^-1^. This mowing speed variation, both in terms of time taken to cut each hectare and time taken to cut each Mg within that hectare, was significantly different between genotypes (*P* < 0.05); particularly between *Mxg* and the other, thinner stemmed varieties. The most important parameter is Mg DM cut h^-1^, OPM 52 and OPM 53 were similar, however, it took 19% less time to cut each Mg of OPM 54 compared to the average of these two and 84% less to cut each Mg of *Mxg* (see **Table 5**).

**Table 5:** Cutting to a swath and baling *Miscanthus* open pollinated *Miscanthus sinensis* (OPM 52, 53, 54) and *Mxg* in the Blankney large scale trial, Lincoln, United Kingdom in spring 2015.

OPM^∗^	Average time per 54 m cut run (s)	Standard deviation of time/run (s)	Harvest rate (s Mg^-1^)	Standard deviation rate (s Mg^-1^)	Tukey comparison group for harvest rate (*p* < 0.001)	Area of one run (ha)	Harvest speed (ha h^-1^)	Yield (t ha^-1^)	harvest rate (Mg h^-1^)	Mean bale weight (kg)
OPM-52	45	4.8	295	31.2	a	0.0243	0.52	6.3	12.1	590
OPM-53	42	3.4	273	21.9	a	0.0243	0.48	6.4	13.2	580
OPM-54	42	3.8	237	21.8	b	0.0243	0.48	7.2	15.1	580
*Mxg*	48	5.2	166	16.7	c	0.0243	0.55	12.8	23.3	530

^∗^*Stem diameters for OPM 12, 52, 53, 54 are approximately half those of *Mxg* but with high stem counts m^-*2*^*.

Baling was performed immediately after cutting as the biomass moisture content was below the 15% moisture level to ensure safe storage and enhance biomass fuel quality. In the case of *Mxg*, which does not flower in the United Kingdom in mild winters, it may not be fully senesced in spring and typically has a moisture content of up to 45% before February cutting. Experience has shown that the 15% moisture level can be achieved by merely drying in the swath in the field (Terravesta Ltd., personal communication). If turning is required to dry the crop further it can be achieved at the rate of 2 ha h^-1^ with a 150 horse power tractor and a hay turner. This has the added advantage of reducing the amount of leaf, which reduces ash content and leaches further Cl, N, P, and K from the material to reduce boiler corrosion and ash sintering, detrimental to combustion quality (Iqbal et al., 2017). These losses are accounted for in this study as the actual harvested yield is determined by the total weight of the baled material.

Baling speed with the large 120 × 120 × 240 Hesston baler depends on the quantity of material baled, normally around 35–40 bales h^-1^ in good ground conditions. Straw bales have a density of 140–180 kg m^-3^, with average bale weights of 540 kg. The weight of *Miscanthus* bales varied with genotype with the thinner stemmed genotype being around 580 kg (density = 171 kg m^-3^) and the thicker stemmed *Mxg* 530 kg (density = 157 kg m^-3^), due to energy required to compact the stiffer material. Thus the rate of baling is ∼3 min Mg^-1^ or for the *Mxg* crop with 12.7 Mg (DM) ha^-1^, a rate of 44 min ha^-1^.

The cost for this operation on an *Mxg* crop with a harvest yield of 12.7 Mg (DM) ha^-1^ for large fields using two tractors and flatbed trailers would be £40.68 Mg^-1^. The GHG emission, including fuel use and C embedded in the machinery, is 4.97 kg CO_2_ eq. C Mg^-1^ (**Table 6**).

**Table 6:** Economic and greenhouse gas costs for harvesting pelletizing and transport per Mg *Miscanthus* biomass.

Harvesting and transport option	Cost per Mg	GHG per Mg

	**£**	**kg CO_2_ eq. C Mg^-1^**
Chipping	£28.00	7.27
Baling	£40.68	4.97
Pelletizing (small scale)	£65.00	45.10
Pelletizing (large scale)	£19.50	13.60

	**£ Mg^-1^ mile^-1^**	**g CO_2_ eq. C Mg^-1^ mile^-1^**

Transport chips	£0.12	35.45
Transport bales	£0.07	18.51
Transport pellets	£0.04	11.36

### Pelleting

The energy to mill and pelletize the *Miscanthus* varied between 1.33 and 0.55 kWh kg^-1^, or between 4.79 and 1.98 MJ kg^-1^. This experiment demonstrated that stiffer, thick stemmed genotypes had higher biomass yields and had a higher pellet density but required more energy to pelletize, in particular *Mxg* (OPM-9). *Mxg* required 1.1 kWh kg^-1^ (3.96 MJ) to mill and pelletize, with a pellet density of 650 kg m^-3^ and a moisture content of 6%. The energy used in this lab scale equipment to pelletize *Mxg* represented 22% of the energy content of the pellets, well above the normal 3–10% of commercial systems (Personal communication from Terravesta Ltd. and Blankney Estates Ltd.). Therefore the data produced in our tests is only useful as a relative comparison between hybrids with different stem properties.

The cost of pelletizing *Mxg* in this experiment at current electricity prices in United Kingdom at £0.13 kWh is £143 Mg, for a small scale commercial plant it would be £65 Mg and for a large scale commercial plant is would be £19.5 Mg. If the pelletizing mills use electricity for grinding, which has a C. intensity of 31.9 g CO_2_ eq. C MJ^-1^ (year 2015 average^5^). The C. cost per Mg of *Mxg* pellets for this test is 99 kg CO_2_ eq. C Mg^-1^. For a small scale commercial pelleting plant it is 45.1 kg CO_2_ eq. C Mg^-1^ and for a large scale plant it is 13.6 kg CO_2_ eq. C Mg^-1^ (**Table 6**).

### Transport Costs

In order to store heaped biomass fuel or transport it in enclosures it must be dry to avoid degradation and its volume must be reduced to minimize storage housing required. In addition this densification is necessary for efficient transportation. Chipped *Miscanthus* has a density in kg m^-3^ of 80–100, bales 140–180 and pellets 650–675. Each format limits the quantity of material that can be carried on a truck – trailer in the United Kingdom by volume to a maximum of 38 bales which is ∼21.5 Mg for *Mxg*, whereas in pellet form a maximum legal load of around 35 Mg could be achieved with a 44 Mg gross weight truck. Chips can only be transported a short distance as even the largest bulk carrier would only be able to transport around 11 Mg. Pellets are a preferred fuel format because they are convenient for storage and transport and comply with fuel feeders, burners, and boilers designed for wood pellets with little modification.

In the United Kingdom, a 44 Mg truck costs ∼£1.46 mile to run including fuels costs at the current rate (2016) for an average annual mileage with a GHG emissions cost of 398 g CO_2_ eq. C mile^-1^ considering an average fuel consumption of 8 miles per gallon of diesel. A full load of pellets would be 35 Mg with a transport cost of £0.041 Mg^-1^ mile^-1^ and a GHG cost of 11.36 g CO_2_ eq. C Mg^-1^ mile^-1^. A full load of 38 *Mxg* bales would be 21.5 Mg with a transport cost of £0.068 Mg^-1^ mile^-1^ and a GHG cost of 18.51 g CO_2_ eq. C Mg^-1^ mile^-1^. A full load of chips would be 11 Mg with a transport cost of £0.133 Mg^-1^ mile^-1^ and a GHG cost of 35.45 g CO_2_ eq. C Mg^-1^ mile^-1^. The harvesting, pelletizing and transport costs are summarized in **Table 6**.

A comparison of the economic cost and GHG cost of harvesting by chipping or baling shows that for both a large and small pelleting plant it is cheaper to transport chips but costs more in GHG emissions (**Table 7**). Analysing the distance that it becomes cheaper to transport pellets rather than bales by road shows that up to 400 miles bales are cheaper and have a much lower overall GHG cost (**Table 8**).

**Table 7:** Relative economic and GHG costs of chips and bales of *Mxg* for different scales of pelleting facilities.

	Throughput	Crop	Land area	Catchment		Cost bales		Cost chips	
	Gg y^-1^	ha	%	km^2^	miles	£ Mg^-1^	kg CO_2_ eq. C Mg^-1^	£ Mg^-1^	kg CO_2_ eq. C Mg^-1^
Small scale pelletizing	5	5,000	10	500	4.6	£105.99	50	£93.55	53
Large scale pelleting	1000	100,000	10	10000	20.6	£61.58	19	£49.98	22

**Table 8:** Cost of bales and pellets for different transport distances.

Distance	£ Mg^-1^		kg CO_2_ eq. C Mg^-1^	
Miles	Bale	Pellet	Bale	Pellet
0	40.46	56.81	5.0	21.6
50	43.86	58.86	5.9	22.2
100	47.26	60.91	6.8	22.7
150	50.66	62.96	7.7	23.3
200	54.06	65.01	8.7	23.9
250	57.46	67.06	9.6	24.4
300	60.86	69.11	10.5	25.0
350	64.26	71.16	11.4	25.6
400	67.66	73.21	12.4	26.1

### Seed Based Yield Trials

**FIGURE 3:**
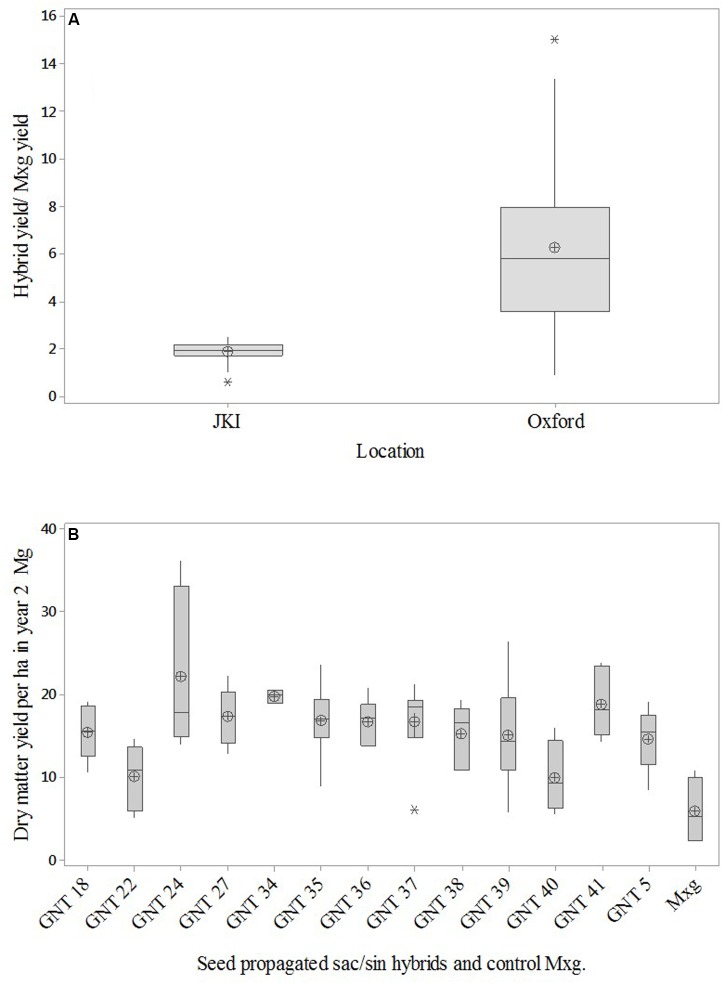
Analysis of second year yield of Elite *Miscanthus sinensis – Miscanthus sacchariflorus* (GNT.xx) seed propagated hybrids compared to *Miscanthus x giganteus* (*Mxg*) rhizome propagated plant in Oxford and Germany (JKI). Box plots of the ratio of GNT yield/*Mxg* yield in year 2 is shown in **(A)** for both locations with the mean (⊕). The individual second year GNTxx and *Mxg* yields are shown in box plot **(B)** with the mean (⊕).

The GIANT Elite seed trials results are not reported here in detail but preliminary results show that all the seed based hybrids, produced by the breeding operations at Aberystwyth (Private communication R. Shafei, Aberystwyth), had yields after 2 years growth that were significantly (ANOVA Tukey test *P* < 0.05) greater than *Mxg* in both Oxford and JKI (**Figure 3B**). The hybrid yields at year 2 are at the level of *Mxg* yield normally reached by year 3 as shown in the OPTIMISC trial (Kalinina et al., 2017). However, the *Mxg* yields in both trials were similar in year 2. The ratio of the seed hybrid yields to *Mxg* yield show mean ratio of 1.9 (*SD* = 0.4) for JKI and 6.3 (*SD* = 3.2) for Oxford (**Figure 3A**), indicating that seed hybrids could also reduce establishment time. These results enabled a farm profitability estimation with the assumption that seed propagated hybrids have yields greater than or equal to *Mxg* yields to estimate the profitability of seed-plug propagation.

### Farm Profitability

**FIGURE 4:**
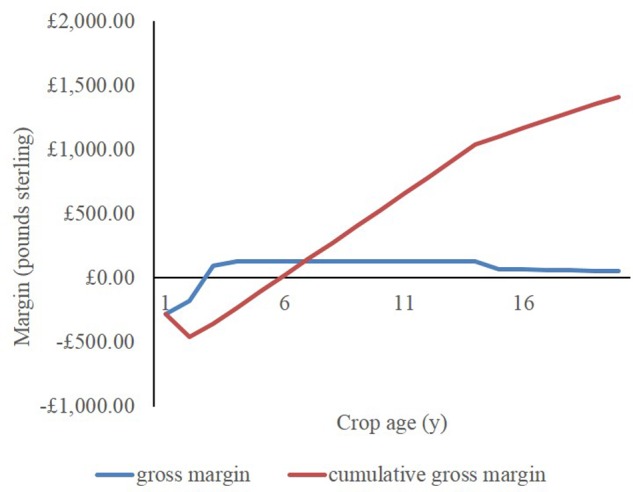
Economic model showing cumulative gross margin or Net Present Value (NPV) per hectare of *Miscanthus* crop for 20 years at Penglais, Aberystwyth using rhizome establishment without film and harvesting by cutting and baling. Simulations uses a 6% discount rate and yields are from the Penglais commercial-scale *Mxg* trial projected for 20 years. The first year harvest is mulched and not sold. *Mxg* bale sale price is £65.00 Mg^-1^.

The longest break-even period and highest cost analyzed here (in Aberystwyth with the lowest yield, slowest establishment, using the most expensive establishment and cut and bale harvesting) was 6 years with a NPV per hectare of £1,331.00 (**Figure 4**). In Aberystwyth film reduces the pay-back time by 1 year and increases the NPV by 12% as the crop achieved maximum yield 1 year earlier. NPV rises to £4,238 with rhizome and film establishment and chip harvesting (**Table 9**). At all locations seed-plug establishment increases NPV by 15% and decreases the payback time by 1 year. Cutting and baling reduces the NPV and increased the payback time by 1 year compared to chipping. In this study, we have considered the impact of crop establishment rates observed in the multi-site, multi-hybrid trials at Aberystwyth, Potash, and Stuttgart which range from 2 to 4 years. The first year crop is mulched and not sold. The plateau yield is estimated as the average yield observed in year 3 and 4 of the trials. Scenarios are summarized in **Table 9** with NPV and break-even year.

**Table 9:** Net present value (NPV) and break-even year of a *Miscanthus* crop per hectare with 20-year crop life, 6% discount rate for different management options, using the costs determined from this study and using a farm gate sale price of £75.0 Mg^-1^ *Miscanthus* crop harvest (moisture < 14%).

Location	Yield establishment ramp				Establishment		Film	Harvesting		NPV	Breakeven
	Year 1	Year 2	Year 3	Year 4+	Rhizome	Seed-plug		Chipping	Baling	£	Year
Aberystwyth	0.3	5.3	12.4	12.4	×			×		£4,238	4
	0.3	5.3	12.4	12.4	×				×	£1,331	6
	0.3	5.3	12.4	12.4		×		×		£5,110	4
	0.3	5.3	12.4	12.4		×			×	£2,533	5
	0.6	9.5	12.4	12.4	×		×	×		£5,229	3
Potash	3.0	9.5	16.0	16.0	×		×	×		£6,469	3
	3.0	9.5	16.0	16.0	×		×		×	£3,096	4
	3.0	9.5	16.0	16.0		×	×	×		£7,515	3
	3.0	9.5	16.0	16.0		×	×		×	£4,142	3
											
Stuttgart	1.0	12.5	12.5	12.5	×		×	×		£4,148	3
	1.0	12.5	12.5	12.5	×		×		×	£1,716	5
	1.0	12.5	12.5	12.5		×	×	×		£5,464	3
	1.0	12.5	12.5	12.5		×	×		×	£2,763	4

## Discussion

This paper reports on measurements of the energy-use and costs involved in the cultivation of *Miscanthus* measured on plot and commercial-scale trials for the first time. These provide inputs for scalable economic models and life cycle assessments of GHG emissions. The models show that at current prices of £75 Mg^-1^ (Bales at <15% moisture) *Mxg* from rhizome with slow establishment rates and current United Kingdom yields of 12–14 Mg ha^-1^, the breakeven payback time is 4 years for chipped harvesting and 6 years with bale harvesting. The worst case scenario of NPV in the United Kingdom is competitive with arable rotations. In continental climates with warmer summers, in this study exemplified by Potash in the Ukraine, yields reach 16 Mg ha^-1^ by the third year and breakeven payback time reduces to the third year including the costs of mulch film and seed-plug establishment. Further reductions in establishment costs are needed to increase farmer acceptance of the crop. Technological developments such as direct seeding, the use of film to speed establishment and the development of higher yielding, faster establishing genotypes are all part of an ongoing research program. For our analysis, it should be noted, that we have compiled parallel advances in breeding, agronomy, and fuel processing. The particular hybrids in this study were chosen as the best material practically available from the Aberystwyth breeding program at the time when the trials were set up. The large scale trial in Blankney set-up in 2012 necessarily used open pollinated nursery seed, selected from good parents, as the production of F1 seed was insufficient in 2011. Methods to scale up seed production of F1 seed have developed year on year, and multi-location trials planted 2014 are based on scalable seed produced in field crossing blocks (Clifton-Brown et al., 2016). **Figure 3** shows the significant improvements in establishment rates and early yields in these newer hybrids, which will reduce financial and C. costs in the future.

Currently, the commercial crop of *Mxg* is already economically viable, though it should be noted that the £75 Mg^-1^ price is supported by the CfD that subsidizes biomass used for electricity generation (Department of Energy and Climate Change [DECC], 2013). Our work shows that crop establishment, yield and harvesting method affect the C. cost of *Miscanthus* solid fuel which for baled harvesting is 0.4 g CO_2_ eq. C MJ^-1^ for rhizome establishment and 0.74 g CO_2_ eq. C MJ^-1^ for seed plug establishment. If the harvested biomass is chipped and pelletized, then the emissions rise to 1.2 and 1.6 g CO_2_ eq. C MJ^-1^, respectively. The energy requirements for harvesting and chipping from this study that were used to estimate the GHG emissions are in line with the findings of Meehan et al. (2013). These estimates of GHG emissions for *Miscanthus* fuel confirm the findings of other Life Cycle Assessment (LCA) studies (e.g., Styles and Jones, 2008) and spatial estimates of GHG savings using *Miscanthus* fuel (Hastings et al., 2009). They also confirm that *Miscanthus* has a comparatively small GHG footprint due to its perennial nature, nutrient recycling efficiency and need for less chemical input and soil tillage over its 20-year life-cycle than annual crops (Heaton et al., 2004, 2008; Clifton-Brown et al., 2008; Gelfand et al., 2013; McCalmont et al., 2015a; Milner et al., 2015). In this analysis, we did not consider the GHG flux of soil which was shown to sequester on average in the United Kingdom 0.5 g of C per MJ of Miscanthus derived fuel by McCalmont et al. (2015a). Changes in SOC resulting from the cultivation of *Miscanthus* depend on the previous land use and associated initial SOC. If high carbon soils such as peatland, permanent grassland, and mature forest are avoided and only arable and rotational grassland with mineral soil is used for *Miscanthus* then the mean increase in SOC for the first 20-year crop rotation in the United Kingdom is ∼ 1–1.4 Mg C ha^-1^ y^-1^ (Milner et al., 2015). In spite of ignoring this additional benefit, these GHG cost estimates compare very favorably with coal (33 g CO_2_ eq. C MJ^-1^), North Sea Gas (16), liquefied natural gas (22), and wood chips imported from the United States (4). In addition, although *Miscanthus* production C. cost is only < 1/16 of the GHG cost of natural gas as a fuel (16–22 g CO_2_ eq. C MJ^-1^), it is mostly due to the carbon embedded in the machinery, chemicals and fossil fuel used in its production. As the economy moves away from dependence on these fossil fuels for temperature regulation (heat for glasshouse temperature control or chilling for rhizome storage) or transport, then these GHG costs begin to fall away from bioenergy production. It should be noted, the estimates in this paper do not consider either the potential to sequester C. in the soil nor any impact or ILUC (Hastings et al., 2009).

This work has shown that most genotypes of *Miscanthus* can be pelleted. Variation in stem morphology influences the energy required and the cost of pellet production. Even though pellets are more expensive to produce than bales, they are still a low C. fuel in a convenient format. Pellets require little site management in storage, unlike bales and chips, which require moisture management and between four and five times as much on-site storage and loading space (even for short term storage). The use of *Miscanthus* fuel in bale format is limited as it requires custom made facilities of at least 40 MW for heat, electricity generation or CHP, which are close to the biomass producing areas. Pellets on the other hand are versatile for automatic feeding of both domestic and commercial-scale burners and boilers because they can be remotely stored and blown by vacuum/airstream or augured. In addition these systems can be easily incorporated into existing site infrastructures. Pellets used for heating are currently subsidized in the United Kingdom by the Renewable Heat Initiative (Department of Energy and Climate Change [DECC], 2013). However, a new fuel and boiler standard is required for *Miscanthus* pellets to be used for domestic heat and concerns about air quality due to emissions from biomass boilers need to be addressed by clean burn, filtration and scrubbing technology.

Our programs have developed technology to propagate *Miscanthus* on a commercial-scale by seed sown plugs which enables ramp up rates of planting areas of around 2,000:1, meaning that a hectare of seed production can produce enough seed to plant ∼2,000 ha. This is two orders of magnitude higher than with rhizomes where 1 ha or rhizome production can plant 10–30 ha. We have quantified the economic and practical benefits of using biodegradable mulch films which reduce the risk of crop establishment failure and accelerate the time to economically viable yields by about 1 year. The economic impact of different harvesting methods indicate that the costs vary from £28 to £40 per ton of biomass harvested in the United Kingdom and are similar to those estimated by Lin et al. (2016) in the United States. These harvesting costs are viable with the current farm gate price of £75 ton. However, as the harvesting of *Miscanthus* is in the spring, it can make use of harvesting equipment used for other crops in summer and autumn. There are certainly savings that can be made with the optimization of the utilization of personnel and equipment, especially on large arable farms with a cereal crop – *Miscanthus* mix. It is also worth noting that in this paper we have only considered costs of contract equipment, large farms and or groups of smaller farms cooperating on a regional basis can reduce costs through machinery ownership or sharing.

In this analysis of economic and GHG cost of *Miscanthus* crops, we have not included the cost of fertilization as it not used in current commercial planting. Most European long term trials of up to 10 years have shown little response to N fertilization except in light sandy soils (Clifton-Brown et al., 2008, 2015; Shield et al., 2014), however, Arundale et al. (2014b) showed that N applications on 5-year-old *Mxg* stands increased yields significantly. The application of N and K to replace nutrients exported at harvest is expected, but there is not enough evidence that these are limiting in the United Kingdom, possibly as a result of processes such as N fixation by endophytic bacteria (Farrar et al., 2014) or atmospheric deposition of transport/other pollution (Goulding et al., 1998). More research is required to determine the optimum rates and at that time the economic and GHG costs can be revised spatially at different scales using models and GIS.

Nodal propagation, where sections are cut from green canes and allowed to root, are not considered in this study. This is the standard method to propagate sugarcane and direct stem transplanting of activated stem buds of *Mxg* was successfully tested, although a question remains on the effect of transplanting time and stem density optimization (Scordia et al., 2015), but it was not explicitly tested in this study. Costs for this method can be estimated from the literature from sugarcane (Xue et al., 2015). The GHG emissions for this method were not estimated.

The invasive potential of seed propagated *Miscanthus* hybrids is often raised as a potential issue. In the OPTIMISC trial fertile flowering hybrids were included. All sites were monitored and found little evidence of spread of *Miscanthus* by seed in the area surrounding areas (Kalinina et al., 2017) because volunteer seedlings rarely establish and successfully overwinter. Nonetheless, for the main biomass production regions of northern and continental Europe, we have concentrated recently on non-flowering hybrids for upscaling because these reach higher yields. While the breeding of sterile triploid seeded hybrids remains a long term goal for *Miscanthus* breeders which would completely eliminate any invasive risk in any environment, current low seed set rates need to be overcome by further research into breeding triploid hybrids.

The experience presented here has highlighted areas for continuing improvement including: further development of bespoke farm machinery for direct sowing, plug planting, harvesting *Miscanthus* and its associated agronomy. Critical foci for further research effort to make biomass systems an economic and environmentally sustainable alternative to fossil fuel energy systems include the breeding of faster-establishing high-yielding seed-propagated genotypes that are suitable for different environments. Preliminary results from on-going work in several projects are making further significant improvements through both breeding and novel agronomies. Optimizing machinery to work specifically with *Miscanthus*, optimizing weed control and crop management to reduce inputs will further reduce costs and GHG input. Economies of scale, with a larger cropped area, will create a competitive market for the machinery and products. In an ideal world, a significant proportion of the embedded fossil fuel GHG cost of this production would itself be replaced by renewable sources, further cutting GHG costs of energy crop production.

Finally it is important to note the influence of policy on the rate of acceptance of use of the crop, both from the demand side and from the supply side. The current level of support in the United Kingdom through the RHI and CfD is sufficient to justify a price of £75 per ton at the farm gate, which enables a farmer to make profit. A commensurate demand-side pull is required through a continuation of these or similar policy measures to ensure that the crops, once established, will have a market for their 15 to 20-year life. The United Kingdom government approval in 2016 for the Sustainable Fuel Register^6^ to certify non-wood biomass fuels as suitable for the RHI is perhaps the most recent positive step in this direction.

## Conclusion

• With mulch film agronomy the latest seeded hybrids establish far more quickly with significantly higher early yields (years 1 and 2) compared to commercial *Mxg* in the United Kingdom delivering a breakeven return on investment at least a year earlier.
• *Miscanthus* crop establishment with seeded hybrids via plugs was found to be more GHG intensive than clonal rhizomes where fossil fuels are used to heat glasshouses but even then this cost is small compared to the gains in yield and scalability.
• High multiplication rates (∼×2000) and lower establishment costs (∼75% of rhizome costs) of seed based hybrids remove a significant barrier to producer and market uptake.
• Further optimization of crop establishment with seeds through plugs are ongoing, including breeding improved hybrids leading to higher yields which will improve profitability and reduce GHG emissions per hectare of production.
• As the growing renewable energy sector contributes more to total energy use, the embedded fossil fuel derived GHG costs in feedstock production and transport will be further reduced overall.

## Ethics Statement

This statement applies to the study as the focus is reducing the GHG emissions and mitigation of climate change by reducing the GHG emissions of bioenergy feedstocks.

## Author Contributions

AH made the data analysis; AH, JC-B, MM, JM, and RS wrote and edited the text; JC-B conceived the study; MM and JM made the commercial-scale trial measurements and established the plots; CN, HS, and MW made the large scale plot measurements; CA made the film trials and assisted with the plot trials; KS conducted the *in vitro* cloning and clone selection; JY constructed the economic model; RS conducted the Elite seed trials; and RS, GS, DS, and SC produced the *Miscanthus* seed.

## Conflict of Interest Statement

The authors declare that the research was conducted in the absence of any commercial or financial relationships that could be construed as a potential conflict of interest.

## Footnotes

1 http://www.carbo-biocrop.ac.uk/

2 http://www.elum.ac.uk/

3 http://www.miscanthusbreeding.org/

4 https://optimisc.uni-hohenheim.de/

5 http://gridwatch.co.uk/co2-emissions

6 http://www.sfregister.org/
